# Phoniatric evaluation: relationships between a set of tests and academic difficulties

**DOI:** 10.1016/j.bjorl.2021.10.004

**Published:** 2021-11-15

**Authors:** Carolina Christofani Sian Kencis, Beatriz Cavalcanti de A.C. Novaes

**Affiliations:** Pontifícia Universidade Católica de São Paulo, São Paulo, SP, Brazil

**Keywords:** Phoniatric evaluation, Phoniatrics, Learning disability, Learning disorder

## Abstract

**Introduction:**

Learning disability has a significant relevance and can generate negative social impacts on the future of an individual if not corrected in time. Phoniatrics is an area of otorhinolaryngology responsible for evaluating, diagnosing, and referring these cases for therapy and/or complementing the multidisciplinary diagnosis.

**Objective:**

To evaluate in school-aged children the association between the application of a set of tests used in the phoniatric assessment and complaints of academic difficulties. To identify which tests would show the main association with academic performance when applied individually.

**Methods:**

Blind, cross-sectional study, subdivided into 4 stages. In the 1st stage, 66 children in the third year of elementary school were submitted to the set of tests of skills applied by the phoniatrician; in the 2nd stage, the physician asked the educators about complaints of academic difficulties regarding the students; in the 3rd stage the students’ Portuguese language school grade was obtained and in the 4th stage an analysis was made between the data.

**Results:**

The educator identified complaints of academic difficulties in 27.2% of the 66 students. There was a statistically significant association between academic difficulties and the following tests of skills: dictation of words and pseudowords, dictation of words, dictation of pseudowords, rhyming, phonemic synthesis, and in the reading test; fluency, intonation and comprehension of the text and morals were analyzed in the reading test. In the multivariate logistic regression analysis, the tests selected to comprise the final model, showing greater association with the academic difficulty complaint were: dictation of words, intonation in reading and comprehension of the morals of the story.

**Conclusion:**

The tests identified as the most relevant in identifying academic difficulties were dictation of words, intonation in reading and comprehension of the morals of the story. However, the other tests that were part of the phoniatric consultation allowed the observation of the individual and their functioning abilities, leading to a better assessment of the case, regarding aspects not directly related to the pedagogical one.

## Highlights

• Relationship between tests used in the phoniatric assessment and the complaint of academic difficulties.
• Reading, text comprehension and dictation assessment: statistically significant correlation.
• Allows prioritizing evidence that may be more related to the complaint.
• It does not replace a complete phoniatric assessment.

## Introduction

Learning difficulties have a significant relevance in the family context and can generate negative social impacts on the future of this individual when there is no timely intervention. This difficulty is multifactorial and encompasses a heterogeneous group of factors capable of altering the child's ability to learn, regardless of their neurological conditions, and may be related to school, family or individual factors.^1^

The worldwide prevalence of learning disabilities is estimated at 20% of the school-age population.^2^ In Brazil, a cross-sectional study^3^ showed a prevalence of 7.6% of children with a specific learning disorder and these high rates show the need to identify possible factors that generate learning disorders.

Phoniatrics is one of the medical specialties responsible for evaluating and diagnosing learning-related pathologies. This assessment aims to identify possible changes in morphological, pathological and social aspects that weaken the ability to learn and, consequently, establish more precise approaches that lead to a more effective multidisciplinary assessment and a timely and appropriate therapeutic approach.^4^

Franchi's study^5^ concluded that some tests would have greater relevance when applied in the context of the phoniatric consultation, and observed that the tests of dictation of words and pseudowords, Child Naming Test, the Digit Span Backward, the copying of pictures, syllabic synthesis, phonemic synthesis, rhyming and phonemic manipulation, associated with reading assessment, and together with data from the clinical phoniatric assessment, would allow the differentiation of subjects with learning difficulties in reading and writing.

Nevertheless, to date, no study has been dedicated to evaluating these tests together and related to academic performance from the educator’s perspective.

## Objective

The objective of this study was to evaluate, in children attending the third grade of Elementary School, a set of tests used in clinical phoniatric assessment, aiming at identifying children with academic difficulties. Moreover, the objective was to determine which of these tests would show a greater association with the clinical complaint of poor academic performance, when applied alone.

## Methods

The research was submitted and approved by the Institution’s Research Ethics Committee under N. 3,727,709 and had the written authorization of the children’s parents or guardians who signed the Free and Informed Consent Form, as well as the children’s consent to participate in the research.

For this investigation, students attending the third grade of Elementary School in a private school were selected. Volunteers with any degree of hearing loss, visual acuity alterations not corrected by the use of refractive lenses, or with known comorbidities that could cause delay in neuromotor development were excluded. The subjects were aged between 8 years and 3 months and 9 years and 10 months, with a mean of 8.2 and a median of 8 years. After the inclusion and exclusion criteria were applied, 66 volunteers were included in the study.

A cross-sectional study was carried out, which was segmented into 4 (four) stages. The examiner, at the time of the phoniatric assessment, did not have any information about the academic performance of each subject. Likewise, the teacher, at the time of the interview, had no information about the student's performance in the tests applied by the phoniatrician.

### 1st stage: application of the tests by a phoniatrician

The selected students were individually assessed by a phoniatrician, at the school, in a quiet room, during regular class time, in sessions lasting approximately 30 min. There was no pre-selection of students regarding the presence or absence of academic difficulties. Students who met the inclusion criteria were consecutively called by name for the phoniatric assessment, following the alphabetical order of the attendance sheet provided by the teachers.

Initially, the professional introduced themselves to the student and, after a brief conversation, informed them that activities related to reading and writing would be carried out. Only after this initial presentation, the battery of tests was started.

The selected tests were those proposed at the conclusion of Franchi’s work (2019), considered the most relevant in identifying a child with academic difficulties, which are: syllabic synthesis, phonemic synthesis, rhyming and phonemic manipulation (which are subtests of the phonological awareness test),^6^ Digit Span Backward,^7^ picture copying,^8^ child naming test,^9^ reading task with retelling and dictation of words and pseudowords.^10^

The order in which the tests were performed was designed by the examiner aiming to prevent the child from showing disinterest or fatigue. Attached (Appendix 1) is the script of the Assessment Form of the activities, shown in the order in which it was followed. Appendix 2 shows the description and illustration of each test.

### 2nd stage

After the subjects’ clinical evaluation, comprising the application of tests and the analysis of the results obtained with the skills tests, an interview was carried out with the teachers, in which information was collected about each student’s academic performance. Each teacher was asked questions aimed at identifying students who, according to the teacher's view, had academic difficulties and whether these difficulties were specifically related to grammar/orthography, text comprehension and issues related to inappropriate behavior in the school environment.

### 3rd stage

The pedagogical coordinator, responsible for the school year in which these students were enrolled, provided each student's grades for the academic period in which this study took place. The grade in the Portuguese language was not related to the work, but to the course syllabus corresponding to the year in which they were enrolled.

### 4th stage

**Figure 1:**
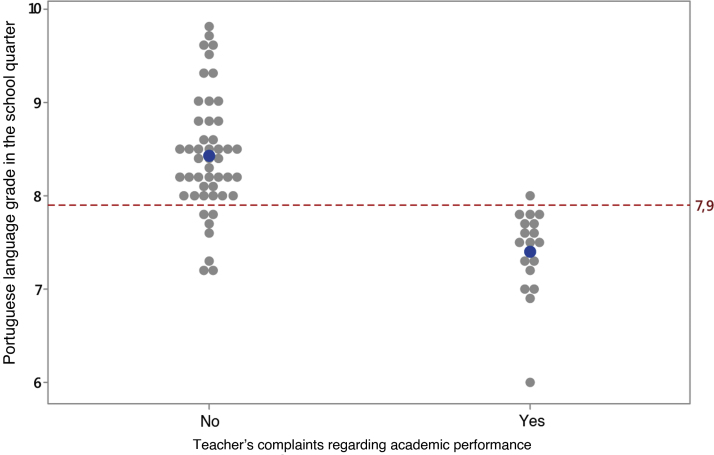
Individual and average values in the Portuguese language grade in the school quarter in the groups with and without teacher complaints. The Student's *t* test showed that the mean grade in the population of those who do not have complaints is higher than in those with complaints (t = 7.22; gl = 40; *p* < 0.001).

**Figure 2:**
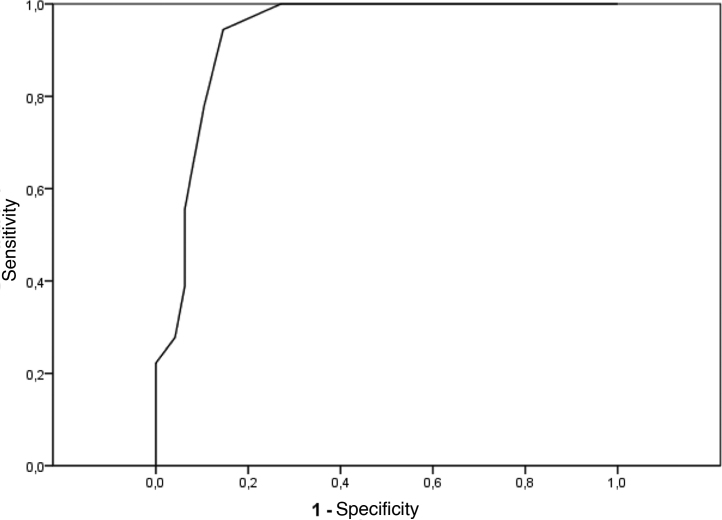
ROC curve obtained by establishing the cutoff value of the Portuguese language grade in the school quarter for the classification of students into groups with and without teacher’s complaints.

The validation of the subjects’ classification took place through the analysis using Student’s *t* test, where agreement was observed between the teacher's opinion, obtained during the interview conducted by the phoniatrician, and the Portuguese language grades during the school quarter (Fig. 1); thus, for analysis purposes, we chose to use the classification by grades. It is important to point out that, in this school, the minimum grade to be approved is 7.0; this fact guided the students’ classification, according to the grade that, after statistical analysis of the data from the ROC curve,^11^ became 7.9 (Fig. 2).

### Data analysis and statistics

The likelihood analysis was carried out individually between the tests applied by the phoniatrician, the presence of academic difficulties, and the three variables identified by the educator when the presence of academic difficulties was identified. Having observed the relationship between the presence of academic difficulties and school grades in Portuguese language, the latter was chosen for data analysis.

To obtain the optimal cutoff value of the Portuguese language grade in the school quarter, aiming to obtain the highest positive predictive value of the student’s classification in the groups with or without complaints of difficulty in reading and writing, according to the teacher, the sample subjects were reclassified into two groups: (1) grade ≤ the cutoff value; or (2) grade > the cutoff value. These groups were used to create a categorized variable (“school grade”), which was equivalent to the academic difficulty complaint.

The value, obtained statistically from the ROC curve study, was 7.9 (Fig. 2) and this variable was then considered in the rest of the analysis. To perform the multivariate assessment of the association of the applied tests and the Portuguese language grade in the school quarter, it was adjusted in a logistic regression model and a significance level was set at 0.05 in the hypothesis tests.

## Results

Table 1 shows the teacher’s opinion regarding the presence of complaints about academic difficulties in reading and writing, and the average Portuguese language grade in the school quarter. Of the 66 evaluated students, the teacher identified academic difficulty in 18 (27.3%) students and the average grade in this group was 7.4, whereas for the 48 (72.7%) students classified without difficulty, the average grade was 8.4 (Table 1).

**Table 1:** Descriptive summary of the Portuguese language grade in the school quarter, in the groups with and without teachers’ complaints.

Complaint	N	Mean	Standard deviation	Minimum	Median	Maximum
No	48	8.4	0.63	7.2	8.4	9.8
Yes	18	7.4	0.47	6.0	7.5	8.0
Total	66	8.1	0.74	6.0	8.2	9.8

To test the existence of an association between the presence of academic difficulties, here categorized as a grade lower than 7.9 in the Portuguese language, the complaints identified by the teacher and the tests applied by the phoniatrician, the likelihood ratio test was used (Fisher and Van Belle, 1993),^12^ which are described below.

### Dictation of Words and Pseudowords

Sixty children (90.9%) had a score ≥ the mean and < the mean. The frequency and percentage distributions in relation to the grade and grammar/orthography and text comprehension complaints are shown in Table 2, Table 3, respectively. There is an association between performance in the word and pseudoword dictation test (total) and the grade in the Portuguese language (*p* = 0.013), and also between grammar and orthography complaints (*p* = 0.002). There is no evidence of an association between dictation performance and text comprehension (*p* = 0.191) and behavior complaints (*p* = 0.108).

**Table 2:** Frequencies and percentages of the Portuguese language grade in the school quarter, in each response category in the dictation test (total). Likelihood Ratio Test: χ_1_^2^ = 6.3, *p* = 0.013.

DICTATION	GRADE		Total
	≤7.9	>7.9	
<Mean	5	1	6
	83.3%	16.7%	100.0%
≥Mean	19	41	60
	31.7%	68.3%	100.0%
Total	24	42	66
	36.4%	63.6%	100.0%

**Table 3:** Frequencies and percentages of the occurrence of complaints in grammar and orthography in each response category in the dictation test (total). Likelihood Ratio Test: χ_1_^2^ = 9.2, *p* = 0.002.

Dictation	Grammar/ orthography complaint		Total
	≤7.9	>7.9	
<Mean	1	5	6
	16.7%	83.3%	100.0%
≥Mean	47	13	60
	78.3%	21.7%	100.0%
Total	48	18	66
	72.7%	27.3%	100.0%

### Word Dictation test

Eight (12%) students performed below average. Table 4, Table 5, Table 6 indicate that there is an association between performance in the word dictation test and grade in the Portuguese language (*p* = 0.001), as well as between grammar/orthography (*p* = 0.001) and behavior complaints (*p* = 0.026). However, there is no significant association between this test and the complaint, reported by the teacher, in text comprehension (*p* = 0.65).

**Table 4:** Frequencies and percentages of the Portuguese language grade in the school quarter, in each response category, in the word dictation test. Likelihood Ratio Test: χ_1_^2^ = 10.3, *p* = 0.001.

Word Dictation	Nota		Total
	≤7.9	>7.9	
<Mean	7	1	8
	87.5%	12.5%	100.0%
≥Mean	17	41	58
	29.3%	70.7%	100.0%
Total	24	42	66
	36.4%	63.6%	100.0%

**Table 5:** Frequencies and percentages of the occurrence of grammar and orthography complaints in each response category in the word dictation test. Likelihood Ratio Test: χ_1_^2^ = 14.97, *p* < 0.001.

Word dictation	Grammar/orthography complaints		Total
	No	Yes	
<Mean	1	7	8
	12.5%	87.5%	100.0%
≥Mean	47	11	58
	81.0%	19.0%	100.0%
Total	48	18	66
	72.7%	27.3%	100.0%

**Table 6:** Frequencies and percentages of the occurrence of behavioral complaints in each response category in the word dictation test. Likelihood Ratio Test: χ_1_^2^ = 4.95, *p* = 0.026.

Word dictation	Behavioral complaints		Total
	No	Yes	
<Mean	5	3	8
	62.5%	37.5%	100.0%
≥Mean	54	4	58
	93.1%	6.9%	100.0%
Total	59	7	66
	89.4%	10.6%	100.0%

### Pseudoword Dictation test

Only 4 (6%) students performed below average. The analysis performed indicates that there is an association between the pseudoword dictation test result with the grade in the Portuguese language (*p* = 0.003) (Table 7) and with the grammar/orthography complaint (*p* = 0.001) (Table 8). However, there was no statistically significant relationship in the test of association between pseudoword dictation and the complaint in text comprehension, and the behavioral complaint reported by the teacher (*p* = 0.069 and *p* = 0.39, respectively).

**Table 7:** Frequencies and percentages of the Portuguese language grade in the school quarter in each response category in the pseudoword dictation test. Likelihood Ratio Test: χ_1_^2^ = 8.55, *p* = 0.003.

Pseudoword dictation	Grade		Total
	≤7.9	>7.9	
<Mean	4	0	4
	100.0%	0.0%	100.0%
≥Mean	20	42	62
	32.3%	67.7%	100.0%
Total	24	42	66
	36.4%	63.6%	100.0%

**Table 8:** Frequencies and percentages of the occurrence of grammar and orthography complaints in each response category in the pseudoword dictation test. Likelihood Ratio Test: χ_1_^2^ = 11.11, *p* = 0.001.

Pseudoword dictation	Grammar/orthography		Total
	No	Yes	
<Mean	0	4	4
	0.0%	100.0%	100.0%
≥Mean	48	14	62
	77.4%	22.6%	100.0%
Total	48	18	66
	72.7%	27.3%	100.0%

### Rhyming

Six (9%) students performed below average. After the analysis, a significant association was observed between the result in the rhyming test with the grade in the Portuguese language (*p* = 0.013) (Table 9) and with the grammar/orthography complaint (*p* = 0.002) (Table 10). There was no association between performance on the rhyming test and complaints of text comprehension (*p* = 0.19) and behavior (*p* = 0.63).

**Table 9:** Frequencies and percentages of the Portuguese language grade in the school quarter in each response category in the rhyming test. Likelihood Ratio Test: χ_1_^2^ = 6.20, *p* = 0.013.

Rhyming	Grade		Total
	≤7.9	>7.9	
<Mean	5	1	6
	83.3%	16.7%	100.0%
≥Mean	19	41	60
	31.7%	68.3%	100.0%
Total	24	42	66
	36.4%	63.6%	100.0%

**Table 10:** Frequencies and percentages of the occurrence of grammar and orthography complaints in each response category in the rhyming test. Likelihood Ratio Test: χ_1_^2^ = 9.22, *p* = 0.002.

Rhyming	Grammar/orthography		Total
	No	Yes	
<Mean	1	5	6
	16.7%	83.3%	100.0%
≥Mean	47	13	60
	78.3%	21.7%	100.0%
Total	48	18	66
	72.7%	27.3%	100.0%

### Phonemic synthesis

The performance of seven (10.6%) students was considered to be below average. After the analysis, it was observed that there is a statistically significant association between phonemic synthesis performance and the Portuguese language grade (*p* = 0.046) (Table 11) and there is no evidence of association with complaints in grammar/orthography (*p* = 0, 07), text comprehension (*p* = 0.26) and behavior (*p* = 0.74).

**Table 11:** Frequencies and percentages of the Portuguese language grade in the school quarter in each response category in the phonemic synthesis test. Likelihood Ratio Test: χ_1_^2^ = 4.00, *p* = 0.046.

Phonemic synthesis	Grade		Total
	≤7.9	>7.9	
<Mean	5	2	7
	71.4%	28.6%	100.0%
≥Mean	19	40	59
	32.2%	67.8%	100.0%
Total	24	42	66
	36.4%	63.6%	100.0%

### Reading assessment

#### Reading fluency

Seven (10.6%) students performed below average. Table 12 shows the statistical significance between this item and the grade obtained at school (*p* = 0.046), as well as Table 13, Table 14, in relation to complaints of difficulty in grammar/orthography (*p* = 0.009) and text comprehension (*p* = 0.003), respectively.

**Table 12:** Frequencies and percentages of the Portuguese language grade in the school quarter in each response category in the reading fluency test. Likelihood Ratio Test: χ_1_^2^ = 4.00, *p* = 0.046.

Reading fluency	Grade		Total
	≤7.9	>7.9	
<Mean	5	2	7
	71.4%	28.6%	100.0%
≥Mean	19	40	59
	32.2%	67.8%	100.0%
Total	24	42	66
	36.4%	63.6%	100.0%

**Table 13:** Frequencies and percentages of the occurrence of grammar and orthography complaints in each response category in the reading fluency test. Likelihood Ratio Test: χ_1_^2^ = 6.74, *p* = 0.009.

Reading fluency	Grammar/orthography		Total
	No	Yes	
<Mean	2	5	7
	28.6%	71.4%	100.0%
≥Mean	46	13	59
	78.0%	22.0%	100.0%
Total	48	18	66
	72.7%	27.3%	100.0%

**Table 14:** Frequencies and percentages of the occurrence of complaints in text comprehension in each response category in the reading fluency test. Likelihood Ratio Test: χ_1_^2^ = 8.77, *p* = 0.003.

Reading fluency	Text comprehension		Total
	No	Yes	
<Mean	3	4	7
	42.9%	57.1%	100.0%
≥Mean	54	5	59
	91.5%	8.5%	100.0%
Total	57	9	66
	86.4%	13.6%	100.0%

#### Intonation and punctuation during reading

Thirteen (19.7%) students performed below average. Table 15, Table 16 show statistical significance between this item and the grade in the Portuguese language (*p* = 0.001) and the complaint of difficulty in grammar/orthography (*p* = 0.001), respectively.

**Table 15:** Frequencies and percentages of the Portuguese language grade in the school quarter in each response category in the intonation/ punctuation marks in the reading test. Likelihood Ratio Test: χ_1_^2^ = 16.31, *p* < 0.001.

Intonation/ punctuation marks	Grade		Total
	≤7.9	>7.9	
<Mean	11	2	13
	84.6%	15.4%	100.0%
≥Mean	13	40	53
	24.5%	75.5%	100.0%
Total	24	42	66
	36.4%	63.6%	100.0%

**Table 16:** Frequencies and percentages of the occurrence of grammar and orthography complaints in each response category in the intonation/ punctuation marks in the reading test. Likelihood Ratio Test: χ_1_^2^ = 13.01, *p* < 0.001.

Intonation/ punctuation marks	Grammar/orthography		Total
	No	Yes	
<Mean	4	9	13
	30.8%	69.2%	100.0%
≥Mean	44	9	53
	83.0%	17.0%	100.0%
Total	48	18	66
	72.7%	27.3%	100.0%

#### Comprehension the Morals of the Story

Thirty-four (51.5%) students did not understand it. Table 17 shows the statistical significance between this item and the Portuguese language grade (*p* = 0.003). There was no statistical significance between difficulty in grammar/orthography, text comprehension, behavior and this test.

**Table 17:** Frequencies and percentages of the Portuguese language grade in the school quarter in each response category in the test of Text Morals Comprehension. Likelihood Ratio Test: χ_1_^2^ = 8.62, *p* = 0.003.

Morals Comprehension	Grade		Total
	≤7.9	>7.9	
No	18	16	34
	52.9%	47.1%	100.0%
Yes	6	26	32
	18.8%	81.3%	100.0%
Total	24	42	66
	36.4%	63.6%	100.0%

#### Text comprehension

Seven (10.6%) students did not understand it, 9 (13.7%) understood after the examiner read it, 27 (40.9%) understood the text superficially, and 23 (34.8%) retold the text with details (Table 18). There was no statistical significance regarding the difficulty in grammar/orthography, text comprehension, behavior and this test.

**Table 18:** Frequencies and percentages of the Portuguese language grade in the school quarter in each response category in the text comprehension/retelling test. Likelihood Ratio Test: χ_3_^2^ = 20.76, *p* < 0.001.

Comprehension/retelling	Grade		Total
	≤7.9	>7.9	
No	7	0	7
	100.0%	0.0%	100.0%
After the examiner read	3	6	9
	33.3%	66.7%	100.0%
Superficial	11	16	27
	40.7%	59.3%	100.0%
Details	3	20	23
	13.0%	87.0%	100.0%
Total	24	42	66
	36.4%	63.6%	100.0%
	72.7%	27.3%	100.0%

### Picture copying, child naming and digit span backward tests

Table 19 shows the *p*-value in the likelihood test in relation to all the tests applied by the phoniatrician and the grade in the Portuguese language in the school quarter in which they were evaluated. In this analysis, the tests of copying pictures, child naming and digit span backward obtained a CI < 95% and, therefore, were not directly related to the Portuguese language grade.

**Table 19:** Relationship between the applied tests and the Portuguese language grade.

Applied tests	Likelihood test (*p-*value) and school grade:
Word dictation	0.001
Intonation/punctuation marks in reading	0.001
Comprehension/retelling	0.001
Pseudoword dictation	0.003
Text Morals Comprehension	0.003
Rhyming	0.013
Word and pseudoword dictation	0.013
Phonemic synthesis	0.046
Reading fluency	0.046
Child Naming Test	0.690
Figure Copying Test	0.207
Digit Span Backward	0.238

The Syllabic Synthesis and Phonemic Manipulation tests, which are subtests of the Phonological Awareness Test, were not statistically evaluated, as all subjects had average or above average grades for their age.

In the logistic regression model adjustment, the response variable was academic performance, which in this study is equivalent to the Portuguese language grade categorized as ≤7.9 or >7.9, that is, a binary variable. A grade > 7.9 was considered a reference category. The tests applied by the phoniatrician considered as explanatory variables were those in which a *p*-value < 0.250 was obtained in the assessment of the association of each one of them with the grade, as shown in the previous tables.

Therefore, the tests considered as explanatory variables were: total dictation, word dictation, picture copying, rhyming, phonemic synthesis, reading fluency, intonation/punctuation, comprehension of text morals and digit span backward tests. After the analysis, the tests selected to constitute the final model were: intonation/punctuation, comprehension of morals and word dictation. Estimates of the coefficients, standard errors, odds ratios, 95% confidence intervals of odds ratios, and *p*-values obtained in the coefficient significance tests are shown in Table 20.

**Table 20:** Estimates of coefficients, standard errors, odds ratios, 95% confidence intervals of odds ratios and *p*-values obtained in the significance tests of the coefficients obtained in the logistic regression model adjustment with the Portuguese language grade categorized as ≤7.9 or >7.9 as the response variable (reference category grade >7.9).

Term	Coefficient	Standard Error	Odds ratio^a^	Confidence Interval (95%) for the Odds Ratio	*p-*value
Constant	4.78	1.67			
Intonation/punctuation marks	−2.6	0.96	0.07	(0.01; 0.49)	0.002
Morals comprehension	−1.82	0.73	0.16	(0.04; 0.67)	0.006
Word dictation	−2.75	1.33	0.06	(0.005; 0.873)	0.021

a Odds ratio greater than or equal to the mean in relation to the lower than the mean in the intonation/punctuation marks and word dictation tests; odds ratio of ‘yes’ in relation to ‘no’ in the text morals comprehension.

The other tests initially considered as explanatory variables had no additional contribution to those selected to constitute the final model. The *p*-values obtained in the variable selection process for these tests are shown in Table 21, and the Hosmer-Lemeshow test (Hosmer and Lemeshow, 2013)^13^ indicated the model goodness of fit (*p* = 0.964).

**Table 21:** *P*-values obtained in the variable selection process in the logistic regression model. Hosmer-Lemeshow test indicated a goodness of fit of the model (*p* = 0.964).

Teste	*p*-value
Total dictation	0.849
Figure copying	0.854
Rhyming	0.865
Phonemic synthesis	0.369
Reading fluency	0.567
Digit Span Backward	0.321

## Discussion

The clinical phoniatric assessment of a child who complains of academic difficulties in reading and writing can consist of several tests that assess these aspects. However, the objective is not to specify this assessment in orthographic and grammatical errors, but to analyze how the child performs the function to obtain the desired result in each skill for a given age, in a playful and dynamic way.

This study showed that the average Portuguese language grade in the population of those subjects who do not have academic difficulties was higher than in students with this complaint (t = 7.22; gl = 40; *p* < 0.001). These data become relevant and statistically equivalent to the complaint presented by the educator regarding academic difficulties.

According to the results of this study, a statistically significant relationship was obtained between the complaint of academic difficulty and the following tests: word and pseudoword dictation (total), word dictation, pseudoword dictation, rhyming and phonemic synthesis. In the reading test, all assessed variables were significant (reading fluency, adequate intonation and punctuation during reading, text comprehension and understanding of the morals of the story).

A logistic regression model was adjusted to perform a multivariate assessment of the association between all applied tests and the Portuguese language grade. This feature allows estimating the probability associated with the occurrence of a given event, given a set of explanatory variables. The word dictation, comprehension of the text morals and adequate intonation and punctuation tests during reading were the three tests selected to constitute the final model, as they were more closely associated with the complaint of academic difficulties.

Based on this analysis, the interpretation of the obtained results is that the probability of a student receiving a grade lower than 7.9 in the Portuguese language when they get a grade higher than or equal to the average in the word dictation, understanding of the text morals and intonation/punctuation performance in the reading test alone will be 0.06, 0.16 and 0.07 times respectively. That is, there is a really low chance of poor academic performance when they perform well in these tests carried out during the consultation.

From an educational perspective, learning difficulties reflect an inability or impediment to learning how to read, write, calculate, or to acquire social skills^14^ and the evidence that assesses these skills has been studied and associated with this performance for decades. In the results of neuropsychological assessment processes, the executive functions and metalinguistic skills are the most commonly associated with learning difficulties,^15^ as well as the dictation tests, as identified in the present study.

Related to executive functions, reading comprehension is, for instance, a complex cognitive activity that involves several skills and different forms of processing, among which are attention, working memory and decoding skills.^16^ Children with reading difficulties have alterations in their fluency and problems with text comprehension, due to changes in phonological perception and low capacity to store information in the working memory.^17^

Therefore, while the process of converting letters into sound units is not automated and there is a disturbance in executive functions, reading comprehension will show limitations. As observed in the present study, good reading fluency, text comprehension and, consequently, the morals of the story are closely related, and can directly impact academic performance.

The dictation writing test (short version) by Seabra & Capovilla (2013) is, by definition, a test that assesses writing and uses terms that vary in lexicality, grapheme-phoneme regularity, length and frequency of occurrence in the Portuguese language. It is strongly related to the written language skills and the finding that a subtest of this evaluation, the dictation of words, was one of those selected to constitute the final model, only reinforces what is currently found in the literature.

It is stated in the literature that phonological awareness is one of the most important skills for learning, but only until a certain point in the development, leaving room for other skills (syntactic and semantic awareness) as the child progresses through schooling.^18^, ^19^ Perhaps for this reason, in this study, the subtests that assessed phonological awareness, such as rhyming and phonemic synthesis, albeit statistically significant, were not relevant enough to constitute the final model tests proposed here, not showing a greater odds ratio when compared to the others.

During academic learning, the information processing depends on the integration of several skills, especially attentional-cognitive, memory and linguistic skills, in addition to emotional and behavioral development.^20^ Therefore, although some tests such as the child naming test and picture copying test have not been classified as statistically significant in relation to the academic difficulty complaint, they constitute tests that show functional oral, visual-motor and visual perception skills, which may contribute to the investigation of other aspects of this individual during the phoniatric evaluation.

During this study, two individuals who were part of the group with academic difficulties drew our attention: individual 1 showed an altered performance in nine of the twelve evaluated items and, during the clinical phoniatric evaluation, it was observed that they presented important phonological speech alterations; on the other hand, individual 2 showed an altered performance in only two of the twelve items applied by the phoniatrician.

At the second stage, during the interview with the teacher, individual 1 was classified as having overcome their pedagogical difficulties, being praised for their interest in learning and for obtaining a high enough grade to be approved at the Portuguese language discipline, while individual 2, who was classified as having restless and sometimes violent behavior in the school environment, was the only one of the 66 evaluated students to fail the third grade of Elementary School.

Several neuropsychiatric pathologic conditions have a greater risk of poor academic performance due to the impairment of functions and skills necessary for learning. These include attention-deficit hyperactivity disorder, pediatric bipolar disorder, oppositional-defiant disorder, anxiety disorders, and others.^21^ These cases exemplify the contribution of the complete phoniatric assessment and it explains that the isolated application of tests that assess cognitive, visual, auditory and motor skills may not be enough to determine the clinical conduct and management, since these students do not always need exclusively extracurricular support of a pedagogical nature.

In this scenario, it is observed that individual 1 would benefit from speech therapy. Although individual 2 obtained results within the expected range for their age in most of the tests, it can be observed that their behavior could be one of the contributing factors for poor academic performance and that they would benefit from a complete clinical phoniatric evaluation, since the global assessment of the individual would better guide the referral to the appropriate therapy or for multidisciplinary diagnostic complementation.

Currently, up to one-fifth of students may have academic difficulties for several reasons, therefore, it is up to the educator to identify those whose academic development is below that for their age and school year, and it is up to parents and/or guardians to seek professional help in these situations.

Considering the large number of direct referrals to the phoniatrician by educational institutions, knowing the skills that may be more related to the educator's complaint regarding academic difficulties can result in a better understanding of the specific complaints related to this individual. The present study is unprecedented in this area and aims to improve the phoniatrician's understanding of clinical assessment situations that have become increasingly frequent.

Thus, this study did not intend to establish a test protocol to be performed during the phoniatric assessment in patients with learning difficulties, whose extensive interview and detailed physical examination, added to the set of tests that are applied by an otolaryngologist within an area of expertise in phoniatrics, aims to provide individualized guidance, whether for therapeutic or diagnostic purposes.

## Conclusion

The present study showed that, in the multivariate logistic regression analysis, among all the tests performed in the study, those that showed the greatest association with complaints of academic difficulties were: word dictation, comprehension of text morals, intonation and punctuation during reading. The identification of this relationship can better guide the conduct during the medical consultation, but it does not exclude the need for a complete phoniatric assessment.

## Funding

This study was carried out with funding from 10.13039/501100002322CAPES - Coordenação de Aperfeiçoamento de Pessoal de Nível Superior, partial scholarship, and the authors declare no conflicts of interest.

## Conflicts of interest

The authors declare no conflicts of interest.
