# Supplementary material for: Phoniatric evaluation: relationships between a set of tests and academic difficulties
Source: Braz J Otorhinolaryngol. 2021 Nov 15;89(1):104–13. doi: 10.1016/j.bjorl.2021.10.004 (PMC9874320; doi:10.1016/j.bjorl.2021.10.004)

**BJORL-D-21-00194 – Supplementary Material**

**Apêndice 1**

**Apêndice 2**

| **1. Tarefa de repetição de números em ordem inversa (Capellini, Smythe e Silva, 2017):** este teste avalia a memória auditiva de trabalho, nele o examinador após enunciar dois exemplos de como seria a atividade e a criança compreender o comando, fala dígitos com diferença de 1 segundo entre eles, em sequências de dois a seis números,e pede que o entrevistado os repita em ordem inversa. Este teste é padronizado e possui tabela de correção por ano de escolaridade da 1a a 5a série do ensino fundamental, sendo classificado como “adequado” ou “sob atenção” de acordo com o número de acertos. |
| --- |
| **2. Cópia de figuras (Santucci & Pêcheux, 1981):** Este teste avalia a organização espacial no plano gráfico, nele é solicitado ao aluno a cópia de 5 figuras geométricas em folha |
| tamanho A4 no sentido horizontal, baseado no Manual para Exame Psicológico da Criança (1981), que apresenta resultado padronizado esperado por faixa etária de 6 a 14 anos de idade. |
| **3. Prova de consciência fonológica por produção oral (Seabra & Capovilla, 2012):** |
| 3.1 Síntese silábica: A criança deve unir as sílabas falada pelo examinador dizendo qual palavra resulta da união destas. São faladas ao todo 6 palavras, sendo destas as duas primeiras para treino até que a criança compreenda o comando solicitado. Este teste é padronizado e possui valores de correção para crianças de 3 a 14 anos de idade e classificado como: muito baixo, baixo, média, alto ou muito alto. |
| 3.2 Síntese fonêmica: A criança deve unir os fonemas falado pelo examinador dizendo qual palavra resulta da união destes. São verbalizadas ao todo 6 palavras, sendo destas as duas primeiras para treino até que a criança compreenda o comando solicitado. Este teste é padronizado e possui valores de correção para crianças de 3 a 14 anos de idade e é classificado como: muito baixo, baixo, média, alto ou muito alto. |
| 3.3. Rima: Avalia a capacidade da criança em discriminar rimas, são verbalizadas 4 sequências de três palavras cada e a criança deve dizer quais são as duas que terminam com o mesmo som. Este teste é padronizado e possui valores de correção para crianças de 3 a 14 anos de idade e é classificado como: muito baixo, baixo, média, alto ou muito alto. |
| 3.4 Manipulação fonêmica: A criança deve adicionar ou subtrair letras de palavras verbalizadas pelo examinador dizendo qual palavra foi formada a partir desta manipulação. São faladas ao todo 6 palavras, sendo que destas as duas primeiras eram para treino até que a criança compreenda o comando solicitado. Este teste é padronizado e possui valores de correção para crianças de 3 a 14 anos de idade e é classificado como: muito baixo, baixo, média, alto ou muito alto. |
| **4. Avaliação de leitura e escrita:** |
| a) Tarefa de leitura com reconto: Foi entregue ao aluno um texto considerado de dificuldade média por apresentar sentido figurado, escrito em letras maiúsculas do tipo “Calibri” número 14, cujo título era “O Feixe de Varas”, foi solicitado inicialmente que o aluno lesse o texto em voz alta, e depois ele era questionado sobre o que havia entendido do conteúdo lido e se ele havia compreendido a “moral da história”.Caso não conseguisse ler ou compreender a idéia principal do texto, o examinador relizava a leitura para a criança e então solicitava que o aluno tentasse interpretar o que havia entendido.  Durante esta avaliação foi observado também a fluência de leitura, e se o examinado respeitava a pontuação do texto conferindo à leitura a correta entonação da frase. Este teste não é padronizado, e foi atribuído nota de 0 a 10 pelo examinador para cada habilidade descrita acima, sendo 0 (zero) para o pior desempenho e 10 (dez) caso não houvesse nenhum erro na leitura.  A classificação da nota se deu a partir da média das notas de todos os alunos avaliados e foi considerado como classificação dentro da média um desvio padrão acima e um abaixo do valor encontrado, para 2 desvios padrão acima foi atribuído a pontuação “alta”, e foi considerada “baixa” quando encontrado 2 desvios abaixo da média.  Cada aluno foi classificado segundo critérios abaixo: |
| i. Sujeitos que não conseguiram ler o texto. |
| ii. Sujeitos que leram o texto, porém não conseguiram recontar nada do texto após sua própria leitura e nem após a leitura do examinador. |
| iii. Sujeitos que leram o texto e não conseguiram recontar nada do texto após sua própria leitura, no entanto, após a leitura ser realizada pelo examinador, foram capazes de recontar o conteúdo geral do texto, sem recontar os detalhes. |
| iv. Sujeitos que leram o texto, e conseguiram recontar a história sem detalhes após sua própria leitura. |
| v. Sujeitos que leram e conseguiram recontar o conteúdo da história com detalhes. |
| b) Tarefa de ditado de palavras e pseudopalavras – versão reduzida – (Seabra &Capovilla 2012): neste teste é realizado um ditado contendo 36 palavras que são escritas em folha pautada de forma a conter 3 palavras por linha, sendo as duas primeiras palavras de cada linha compostas de palavras existentes e que variam em função de características psicolinguísticas de lexicalidade, frequência, regularidade e comprimento, e a última palavra da linha composta por uma pseudopalavra, no total são 24 palavras e 12 pseudopalavras. O teste tem pontuação padronizada para crianças de 6 a 11 anos de idade, e para a correção das “palavras” são consideradas corretas palavras escritas ortograficamente corretas, e para as “pseudopalavras” são consideradas acertos quando fonologicamente corretas, mesmo que com letras diferentes do gabarito. Classificação do desempenho em: Muito baixo, baixo, média, alto ou muito alto. |
| c) Teste Infantil de Nomeação (Seabra et al, 2012): este teste padronizado avalia a linguagem expressiva e memória de longo prazo de crianças de 3 a 14 anos, o examinado deve nomear verbalmente 60 figuras que lhe são apresentadas, uma a uma. Cada figura nomeada corretamente ou com uma palavra que seja o sinônimo desta é pontuada com 1 ponto. Classificação do desempenho em: Muito baixo, baixo, média, alto ou muito alto. |


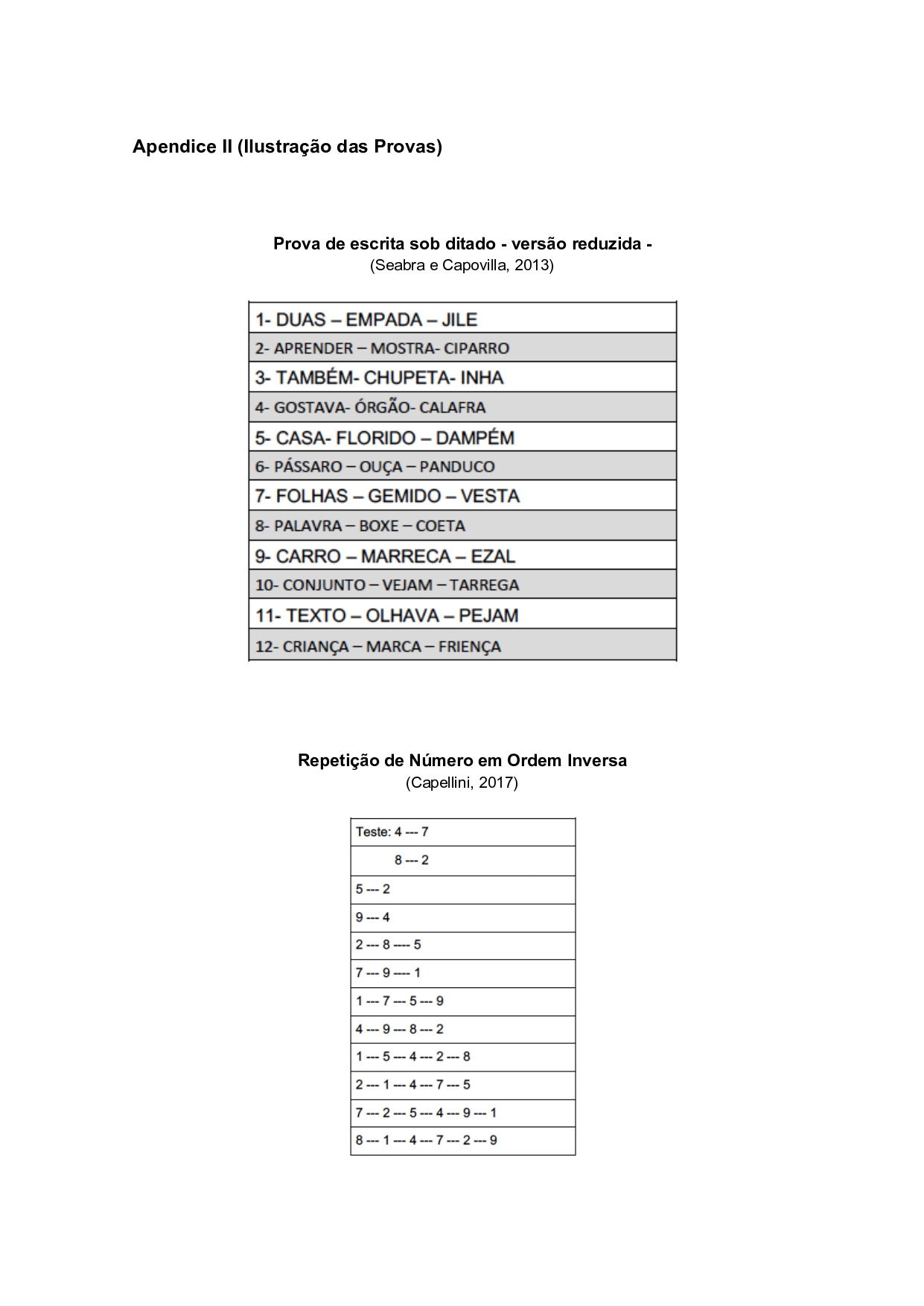


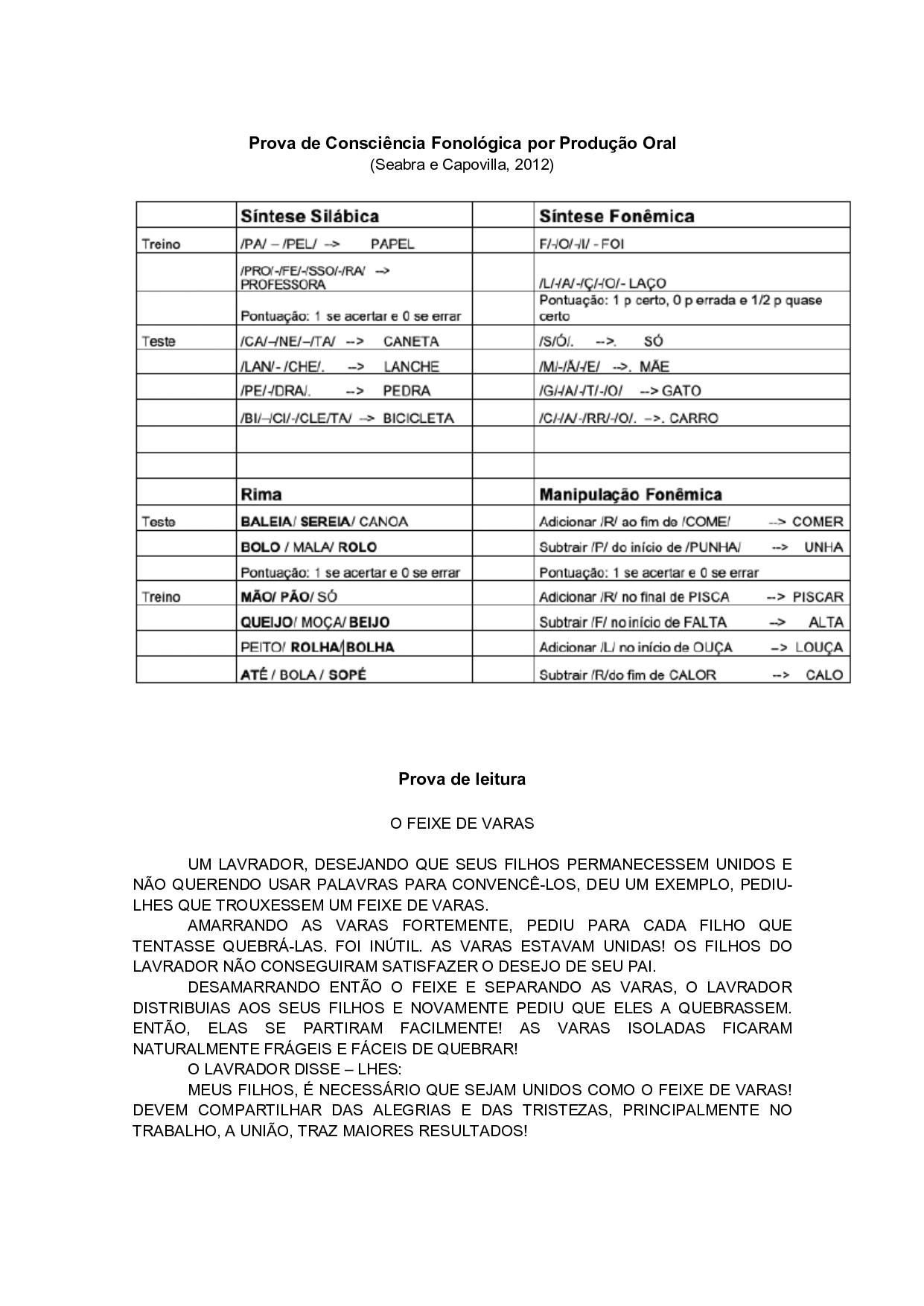

Supplement: Supplementary file 1 [file mmc1.docx]
